# COVID-19 Impact on Diagnosis and Staging of Colorectal Cancer: A Single Tertiary Canadian Oncology Center Experience

**DOI:** 10.3390/curroncol29050268

**Published:** 2022-05-04

**Authors:** Mathias Castonguay, Rola El Sayed, Corentin Richard, Marie-France Vachon, Rami Nassabein, Danielle Charpentier, Mustapha Tehfé

**Affiliations:** 1 Department of Medicine, Université de Montréal, Montreal, QC H3T 1J4, Canada; rola.el.sayed@umontreal.ca (R.E.S.); rami.nassabein@umontreal.ca (R.N.); danielle.charpentier@umontreal.ca (D.C.); 2 Department of Hemato-Oncology, Université de Montréal, Montreal, QC H3T 1J4, Canada; 3 Centre Intégré de Cancérologie du CHUM, Université de Montréal, Montreal, QC H2X 3E4, Canada; marie-france.vachon.chum@ssss.gouv.qc.ca; 4 Centre de Recherche du CHUM, Université de Montréal, Montreal, QC H2X 0A9, Canada; corentin.richard@umontreal.ca

**Keywords:** COVID-19, colorectal cancer, cancer screening, diagnosis

## Abstract

Background: Public health measures have imposed drastic reductions in cancer screening programs at the beginning of the COVID-19 pandemic, with an unknown impact on the diagnosis and staging of colorectal cancer (CRC). Methods: Newly diagnosed CRC cases at the Centre Hospitalier de l’Université de Montréal (CHUM) were divided into two groups according to the timeline: pre-pandemic (1 January 2018–12 March 2020), and pandemic (13 March 2020–30 June 2021) periods. Colonoscopy, surgery, and staging at diagnosis during the pandemic period were compared to the pre-pandemic period. Results: 254 CRC diagnoses were made during the pre-pandemic period in comparison to 125 during the pandemic period. Mean diagnosis rates were lower in the pandemic period (7.8 vs. 9.8 diagnoses/month, *p* = 0.048). Colonoscopy deadlines were less respected in the pandemic period (51.7% vs. 38.3%, *p* = 0.049). The rate of elective surgery did not differ (2.9 vs. 3.5 surgeries/month, *p* = 0.39) and mean delays were similar (58.6 vs. 60.4 days, *p* = 0.77). Stages at diagnosis did not differ (*p* = 0.17). Most of the delayed colonoscopies led to a stage 0 or I CRC (*p* = 0.2). Conclusion: In our center, the COVID-19 pandemic resulted in a decreased rate of CRC diagnosis and increased endoscopic delays without affecting the rate of advanced stage disease. Delays to surgery were quite similar once the CRC diagnosis was established.

## 1. Introduction

The province of Quebec was the epicenter of the COVID-19 pandemic in Canada during the first wave (March to June 2020. Over 55 thousand cases of infection were confirmed [1,2]. In preparation for a possible massive influx of infected individuals requiring medical care, public health authorities imposed drastic reductions on all “non-essential” medical activities including endoscopies, surgeries and variable oncologic screening programs. Those reductions lead to delays in colonoscopy activities that still have not been caught up today.

During the first wave of the pandemic (March to May 2020), the Ministry of Health and Social Services (MSSS) of Quebec reported a 74% reduction of FIT (fecal immunochemical test), a 66% reduction in colonoscopy and a 30% reduction in colorectal cancer (CRC) surgical activities in Quebec, Canada [3]. A recent update from the MSSS reported the recuperation of FIT, colonoscopy, and surgery rates per month to levels comparable to the 2018–2019 period in Quebec. Nevertheless, there was no compensation for the lower rates observed in the first wave. After one year of the pandemic, they still observed a 26% reduction of FIT, 24% reduction in colonoscopy, and 21% reduction in CRC surgical activities [4].

Unfortunately, those observations were not unique to the province of Quebec, as multiple studies across the world have documented similar impacts of the pandemic on health care in general, and CRC screening, diagnosis, and treatment in particular [5,6,7,8,9].

Studies have also tried to create a model-based estimate of the impact of reduced CRC screening on CRC-related diagnosis and mortality. In Canada, He Yong et al. estimated the impact of an interruption of three to six months in screening for CRC using microsimulation models (OncoSim). They found out that 1100–2200 more CRC could be diagnosed due to the progression of adenomas to adenocarcinoma, with over 60% of the cases being at an advanced stage (III or IV) over Canada [10]. Furthermore, in a single center study in Ontario, Canada, L Force et al. found a trend towards higher stage disease in newly diagnosed CRC. However, no statistical significance was reached after six months of the pandemic [11]. In England, Morris et al. estimated from the National Health Services that approximately 3500 patients missed their diagnosis due to pandemic healthcare service restrictions [12].

To our knowledge, no published study has yet documented the impact of the pandemic on colonoscopy and surgery delays, in addition to consequent effects on CRC staging in Quebec after more than one year of the pandemic. We believe that with the COVID-19 pandemic, we will observe longer delays leading to a higher rate of advanced stage disease at diagnosis and thereby worse outcomes in the long term.

In our study, we assess the COVID-19 pandemic impact on the number of newly diagnosed CRC cases in addition to the delays in obtaining requested colonoscopies and surgeries at the CHUM.

## 2. Materials and Methods

This is a retrospective observational, chart-review based unicentric study taking place at the CHUM. All newly diagnosed CRC with an available pathological report at the CHUM within the period from 1 January 2018, to 30 June 2021, were found using the SARDO registry, which represents our institution’s oncology data archiving system. Non-adenocarcinoma cases were excluded. Endoscopic treatment reference (ex: endoscopic mucosal resection) from other centers were excluded. Relapses, defined as locoregional or metastatic recurrence (clinically or radiologically) of a diagnosed CRC after an initial response to treatment, were excluded [13]. Thereby, only newly diagnosed cases of CRC investigated following symptomatic presentation, or a screening program were analyzed. Chart reviewing occurred from November 2020 to August 2021 and data regarding colonoscopy, surgery and staging at diagnosis were obtained and verified retrospectively. Patients were divided into two groups depending on the date of diagnosis: the pre-pandemic period dating from 1 January 2018, to 12 March 2020, versus the pandemic period from 13 March 2020, to 30 June 2021. Of note, 13 March 2020, was chosen as the cutoff date as it represents the date of the institution of restrictions on cancer screening programs by provincial officials in Quebec, Canada.

**Figure 1:**
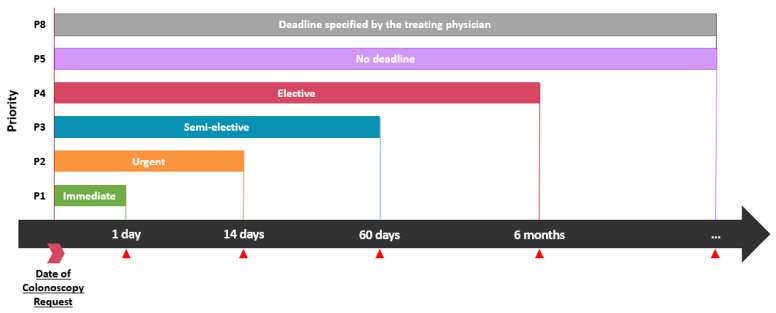
Ministry of Health and Social Services (MSSS) grading system for elective colonoscopy in Quebec, Canada.

Only the priority of the first colonoscopy request was analyzed. Failure to attain a biopsy during the initial colonoscopy would necessitate the request of an alternate colonoscopy with a higher priority. Such requests were excluded to avoid confusion in data analysis. Priority of elective colonoscopy was defined using the MSSS grading system ranging from P1 to P5 [14]. Figure 1 helps to better understand the required time to perform elective colonoscopy depending on priority. P1 colonoscopies are performed within 24 h and are indicated for urgent and possibly life-threatening situations such as heavy lower gastrointestinal bleeding. P2 colonoscopies should be performed in less than 14 days; they are indicated for clinically suspected CRC (based on imaging studies, previous colonoscopies, or physician-based clinical judgment). P3, to be performed in less than 60 days, are mostly indicated for a positive FIT, iron deficiency anemia or rectal bleeding. P4 colonoscopies should be performed in less than six months and are indicated for a change in stool habits or rectal bleeding in young individuals less than 40 years of age. P5 colonoscopies are performed as screening and do not have deadlines. P8 priority indicates a specific deadline required by the treating physician.

Regarding the staging of non-metastatic disease, pathology reports from surgical specimens were used if no neoadjuvant treatment was received. For rectal cancer, pelvic MRI (magnetic resonance imaging) at diagnosis was used if neoadjuvant treatment were received. For metastatic disease at diagnosis, imaging reports from CT scans or PET scans were used. The TNM staging of CRC was defined using the AJCC UICC 8th edition.

We compared delays to colonoscopy according to priority, delays to surgery according to the date of the biopsy, and the difference in CRC staging at diagnosis between the pandemic and the pre-pandemic periods using t tests and Chi-square tests as appropriate using statistical software program R. All tests were two-sided and a *p*-value ˂ 0.05 was considered as significant. Only delays in elective surgery intended as curative treatment were analyzed, excluding surgery performed after neoadjuvant treatment (mostly stages II and III rectal cancers) or for urgent conditions such as intestinal occlusion. Cases with incomplete CRC staging (patients who deferred further investigation or treatment) or missing initial colonoscopy requests (performed in peripheral centers) were kept in the absolute number of CRC diagnosed but excluded from further analyses.

Approval from the ethic and research committees were obtained in November 2020 (approval number 20.255), with a waiver of consent obtained due to minimal risk associated with the study of retrospective design. No funding was received.

## 3. Results

The CHUM has one of the most active oncology centers in Quebec, Canada. Approximately 100 to 130 new CRC are diagnosed annually.

**Figure 2:**
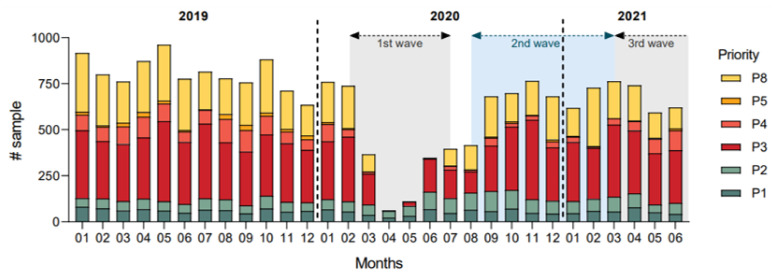
Colonoscopies performed at the CHUM per month with waves of the COVID-19 pandemic in Quebec, Canada. Waves of the pandemic in Quebec: 1st wave from 25 February 2020, to 11 July 2020; 2nd wave from 23 August 2020, to 20 March 2021; and 3rd wave from 21 March 2021, to 17 July 2021 [15].

Figure 2 represents the number of colonoscopies performed at the CHUM per month. Severe restrictions were observed during the first wave, with a return to normal rate per month around September 2020. Less severe restrictions were observed at the end of the second wave (January 2021) and during the third wave (May 2021). No increased endoscopic activity was observed to compensate initial restrictions. P3, P4 and P5 colonoscopies seem to be the most affected whereas P1 and P2 colonoscopies are stable over months.

Table 1 describes the clinical characteristics of patients diagnosed with a new CRC. During the pre-pandemic period (26 months) and the pandemic period (16 months), 254 and 125 CRC diagnoses were made, respectively. The median age at diagnosis was 67.5 years old in the pandemic period compared to 69.4 years old in the pre-pandemic period (*p* = 0.17). The male-to-female ratio of the pandemic period differed from the pre-pandemic period, 0.87:1 compared to 1.40:1 (*p* = 0.03). Colon cancers represented 76.8% of CRC cases in the pandemic period compared to 58.7% in the pre-pandemic period (*p* < 0.001).

### 3.1. Colorectal Cancer Diagnosis Rates

Mean diagnostic rates during the pandemic period tended to be lower than the pre-pandemic period (7.8 vs. 9.8 diagnoses per month, *p* = 0.048). In the pandemic period, 35 patients (37.9%) had a diagnosis of CRC during hospitalization compared to 43 patients (25.9%) during the pre-pandemic period (*p* = 0.048). However, inpatient diagnosis rates per-month did not differ (2.2 vs. 1.7 diagnoses per month, *p* = 0.27). Reasons for hospital admission were quite variable including iron deficiency anemia, infection, intestinal occlusion secondary to cancer, etc. No predominant cause was observed in either group.

### 3.2. Colonoscopy Priority and Delays

In the pandemic period, 51.7% of elective colonoscopies leading to a diagnosis of CRC did not meet the required delays prespecified by request-imposed priorities, compared to 38.3% in the pre-pandemic period (*p* = 0.049). P3 colonoscopies were the most affected (106.5 vs. 58.9 days, *p* < 0.001). P2 colonoscopies did not experience an increase in delays (25.2 vs. 20.9 days, *p* = 0.39). Furthermore, there was no difference in delays of P1 colonoscopy (*p* = 0.89). However, less P1 colonoscopies were performed (11 vs. 1, *p* < 0.001). No P4 or P5 colonoscopies leading to CRC diagnosis were performed during the pandemic, compared to 10 colonoscopies performed before 13 March 2020.

### 3.3. Surgery and Delays

Overall, 2.9 elective curative surgeries per month were performed during the pandemic period, compared to 3.5 in the pre-pandemic period (*p* = 0.39). There was no absolute difference in delays to surgery once the biopsy was obtained (58.6 vs. 60.4 days, *p* = 0.77). When analyzed per-year, no differences in delays were found (*p* = 0.31).

### 3.4. Colorectal Cancer Staging at Diagnosis

**Figure 3:**
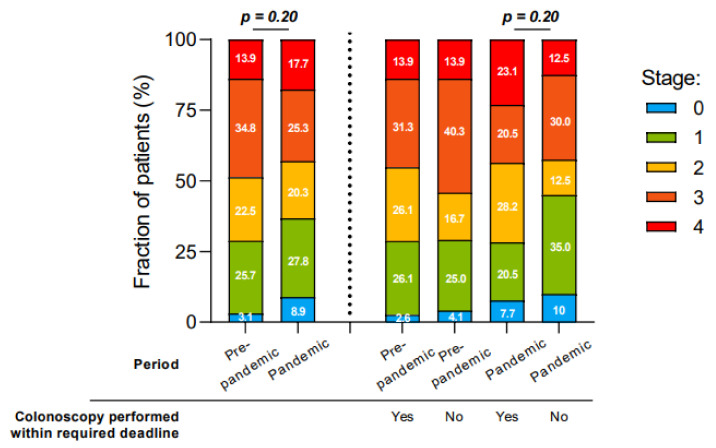
Impact of the COVID-19 pandemic on staging of CRC at diagnosis.

In our center, disease stage at diagnosis did not differ from the pre-pandemic period to the pandemic period as shown in Table 2 (*p* = 0.17). Figure 3 elaborates the different CRC diagnostic staging in correlation with colonoscopy delays (dependent on predefined deadlines). Only patients with a known priority of elective colonoscopy and staging at diagnosis were used for this analysis. Staging at diagnosis was similar among patients in both pre- and pandemic periods regardless of whether the colonoscopy was performed within or beyond the predefined deadline. Most of the delayed colonoscopies abutted in stage 0 or 1 CRC diagnoses (*p* = 0.2).

## 4. Discussion

Despite the concordance of patient characteristics in both studied groups (pre- and per-pandemic), we noticed a tendency to a lower proportion of men diagnosed with CRC, during the COVID-19 pandemic. Several studies have demonstrated that men are less likely to seek medical attention, more likely to delay consulting a health professional and more likely to minimize health issues [16,17]. This could possibly partially explain this observation, however further studies with bigger patient samples are needed to prove such observation.

Furthermore, an increase in colonoscopy delays beyond the MSSS defined deadline was noted, especially for the P3 priority category, with a mean increase of 47.6 days during the pandemic period. As P3 colonoscopies are mostly indicated for asymptomatic or minimally symptomatic patients, they tend to lead to stage 0 or 1 CRC, and that did not differ from the pre-pandemic period. No higher rate of advanced stage disease was observed among patients whom colonoscopy was delayed in both groups. Patients with severe symptoms suggestive of a higher stage CRC were hospitalized or prioritized as P1 or P2 colonoscopies and no increase of delays nor a higher rate of advanced stage disease was observed.

A possible explanation of the latter lies with the known natural history and progression of CRC. It is a heterogeneous disease, with adenomas leading to adenocarcinomas usually over several years (10–15 years) and then progressing at variable rates depending on cancer genomics and patient factors [18,19]. Time to progression of low-stage disease to higher stage disease is highly variable. Hisabe et al. demonstrated that tumor doubling time is inversely proportionate to tumor invasion and may be as long as several months (4.7 to 12.2 months) for invasive tumors [20]. Therefore, we believe that a mean augmentation of 50 days may not be enough to observe a higher rate of advanced stage disease at a population level. However, even if overall staging at diagnosis is similar, we did not address whether tumor size differ. In Ontario, Canada, L Force et al. noticed that CRC operated during the pandemic had a larger tumor size and may represent a shift toward disease severity [11].

In our study, the overall diagnosis rate per month tended to be lower in the pandemic period. Indirectly that could imply that some CRC diagnoses may probably have been missed. Fewer CRC diagnoses were made in a screening process as corroborated with the decrease of P3, P4, and P5 colonoscopy rates during waves of the pandemic observed in Figure 2. Based on the diagnosis rate per month of the pre-pandemic period, we estimate that approximately 30 CRC diagnoses have not been made due to the pandemic. Whether those missed CRC diagnoses will be diagnosed at a higher stage remains unknown. Interestingly, although higher proportion of patients had a diagnosis of CRC during hospitalization in the pandemic period, when comparing the rate of inpatient colonoscopies per month, the difference was insignificant.

These findings are consistent with observations made in other Canadian provinces. For example, in Alberta, Canada, Walker et al. studied how the COVID-19 pandemic affected the diagnostic pathways to CRC. They reported a drastic reduction in the number of diagnoses made post referral due to a positive FIT (asymptomatic screening) or an abnormal lab work within the first six months of the pandemic. As in our study, their rates of symptomatic CRC diagnosis were not affected [21].

The COVID pandemic has had a profound impact on the health system and especially on cancer screening and diagnosis. The restrictions imposed at the beginning of the pandemic are not unique to colorectal cancer investigation. Similar observations were made regarding other cancer sites involving upper endoscopic procedures, bronchoscopy, mammography, and specific prostatic antigen dosing [8,9,10,22]. More precisely in Quebec, an overall decrease of 5% of cancer diagnosis was seen after one year of the COVID-19 pandemic and even after the recuperation of rates of testing for breast and prostate cancer to pre-pandemic levels, no compensation was noted for the period of initial restrictions [4].

We believe that understanding the impact of the reduction of screening programs on diagnosis and treatment is crucial for cancer management in future pandemics. In our opinion even if the rates of advanced stage disease were similar after 16 months of the pandemic, a significant proportion of patients are probably undiagnosed and the overall impact on cancer mortality is difficult to determine. The impact of the COVID-19 imposed restrictions on the overall CRC diagnosis rates, staging, morbidity and mortality needs to be further investigated in the upcoming years.

To limit further COVID-19 impact on CRC screening and diagnosis, several suggestions have been proposed. One of particular interest, is maintaining an organized cancer screening program, independent of in-person clinic visits, that maximizes the use of FIT to prioritize patients to colonoscopy [23]. It has been used in several countries but it needs to be maintained regardless of future pandemic states. Centers in Austria and Michigan, US, use a system where FIT kits have been distributed to homes and returned by mail, demonstrating a great reduction in need of in-person visits [24,25]. As for access to colonoscopy, effort could be made to encourage endoscopic centers to share and distribute patients with abnormal FIT or encourage the implementation of specific centers dedicated to colonoscopy screening without the need for in-hospital visits [26].

Our study has several limitations. Its unicentric nature prohibits any generalization of findings, especially that CHUM is considered as a tertiary referral center, and thereby may be less affected by provincial restrictions [27]. Smaller community hospitals with less flexibility and less staff as well as more saturated health care systems could have experienced a greater impact such as perhaps longer delays in CRC diagnostic procedures. Furthermore, delays of diagnostic and therapeutic procedures may vary between hospitals depending on the local COVID-19 epidemiology. Second of all, the retrospective chart-review basis of our study presents a significant limitation, noting that a non-negligible proportion of elective colonoscopy requests or staging at diagnosis were missing from our medical software and not available for analysis. However, these numbers are stable over the years and should not affect the interpretation of CRC staging since only CRC with a known colonoscopy priority were analyzed. Finally, our study did not address precancerous lesions. Precancerous lesions such as adenomas could possibly progress to adenocarcinomas and lead to an increase in CRC diagnosis over the next few years.

## 5. Conclusions

In our tertiary Canadian oncologic center, 16 months of the COVID-19 pandemic led to an overall decrease in CRC diagnosis, increased colonoscopy delays without a higher rate of advanced stage disease. Most of the delays occurred for colonoscopies indicated for asymptomatic or mildly symptomatic patients with less performance urgency. No difference in delays was observed for life-threatening symptoms or highly suspected CRC requiring colonoscopy. No higher rate of CRC diagnosed because of severe symptoms was observed. Once the diagnosis of CRC has been established, delays to surgery were similar to the pre-pandemic area. In conclusion, despite a decrease in number of overall colonoscopies, in addition to some delays in performance of colonoscopies, no major impact was noted staging-wise. Nevertheless, it might be too early to judge considering that some CRC are still undiagnosed. Upcoming days will play a major role in demonstrating whether an increase in the number of patients with advanced stages will occur in the near future, thereby leading to increased disease morbidity.

## Figures and Tables

**Table 1:** Clinical characteristics of patients diagnosed with CRC during the pre-pandemic and the pandemic period.

	Pre-Pandemic Period (N = 254)	Pandemic Period (N = 125)	
Median age at diagnosis (years)	69.4	67.5	^α^*p* = 0.17
Sex, n (%)			^β^*p* = 0.03
Male	148 (58)	58 (46)	
Female	106 (42)	67 (54)	
Cancer Localization, n (%)			^β^*p* < 0.001
Colic	149 (58.7)	96 (76.8)	
Rectal	105 (41.3)	29 (23.3)	
Elective colonoscopies MSSS priority			^β^*p* = 0.07
P1	11 (4.3)	1 (0.8)	
P2	73 (28.7)	37 (29.6)	
P3	85 (33.5)	45 (36.0)	
P4	9 (3.5)	0 (0)	
P5	1 (0.3)	0 (0)	
P8	18 (7.1)	4 (3.2)	
Unknown *	13 (5.1)	3 (2.4)	
Colonoscopy performed during an hospitalization	44 (17.3)	35 (28.0)	^β^*p* = 0.02
Colonoscopies indicated for a positive FIT *	39 (15.4)	20 (16.0)	^β^*p* = 0.87

* Fraction is expressed over the number of CRC with a known priority of colonoscopy. α *t* test *p*-value β chi-squared test *p*-value.

**Table 2:** The impact of the COVID-19 pandemic on colonoscopies, surgeries and staging of CRC.

	Pre-Pandemic Period (N = 254)	Pandemic Period (N = 125)	
Diagnosis, per month	9.8	7.8	^α^*p* = 0.048
Diagnosis during hospitalization, n (%)	43 (25.9)	35 (37.9)	^β^*p* = 0.048
Diagnosis during hospitalization, per month	1.7	2.2	^α^*p* = 0.27
Elective colonoscopies exceeding deadline, n (%) *	74 (38.3)	42 (51.7)	^β^*p* = 0.049
Delays of elective colonoscopies, days			
P2	20.9	25.2	^α^*p* = 0.39
P3	58.9	106.5	^α^*p* < 0.001
Surgeries, per month	3.5	2.9	^α^*p* = 0.39
Delays to surgery, days	60.4	58.6	^α^*p* = 0.77
Stage at diagnosis, n (%)			β*p* = 0.17
0	11 (4.4)	7 (5.5)	
I	60 (23.6)	26 (21.0)	
II	56 (22.0)	24 (19.4)	
III	75 (29.5)	29 (23.4)	
IV	41 (16.1)	25 (20.2)	
Unknown	11 (4.4)	13 (10.5)	

* CRC with unknown priority of colonoscopy were removed from subsequent analysis. α t test *p*-value β chi-squared test *p*-value.

## Data Availability

The data presented in this study are available on request to the corresponding authors. All patient data were acquired from our institutional data base as per our ethical and research committee approval. No data was shared with any third party or any public data sets.
